# The Fermi problem: Estimation of potential Billing losses due to Undercoding of Florida Medicare data

**DOI:** 10.1016/j.rcsop.2023.100238

**Published:** 2023-03-06

**Authors:** Andrew Tenpas, Eric Dietrich

**Affiliations:** a Texas A&M Irma Lerma Rangel College of Pharmacy, 1010 W. Avenue B, Kingsville, TX 78363, United States of America; b University of Florida College of Pharmacy, 1225 Center Drive, Gainesville, FL 32610, United States of America

**Keywords:** Pharmacy billing, Medicare, Incident-to, Undercoding, Clinical pharmacists

## Abstract

Billing issues are more commonplace than most healthcare professionals, including pharmacists, even realize. Undercoding—or billing outpatient visits for a lower level of service than may be justified—leads to decreased reimbursement, but almost no data captures what is being sacrificed, especially at the state level. Using publicly available data from the National Ambulatory Medical Care Survey and Centers for Medicare and Medicaid Services, we attempt to approximate just how much Medicare reimbursement is lost annually to undercoding in Florida. We also discuss the hidden dangers of undercoding, including how it could hinder the ability of clinical pharmacists to build sustainable clinical services and contribute to the broader healthcare team.

## 1 Introduction

### 1.1 What is a Fermi Problem?

In July 1945, the physicist Enrico Fermi was in Los Alamos, New Mexico for the detonation of the world's first atomic bomb. One objective of the test was determining the bomb's overall yield. It is rumored that Fermi dropped a few scraps of paper as the blast's shockwave passed by. After some quick and informal calculations, he estimated the overall yield to be 10 kt, though we now know the yield was closer to 20 kt.^1^^,^^2^ It was, in hindsight, an excellent approximation. Whether this story is true or not, it spawned the notion of the “Fermi Problem,” which seeks a fast, rough estimate for a question that is either difficult or impossible to measure directly.^3^ In essence, it provides a ballpark figure until more precise measurements or calculations can be made.^4^

A classic Fermi Problem asks “*How many piano tuners are there in Chicago*?”^5^ To answer this question, one needs certain pieces of information. For example, one might need to know Chicago's total population, the average household size in the city, the average number of households needed to own one piano, how frequently the average piano must be tuned, how long it takes someone to tune a piano, and the average work week of a tuner.^6^^,^^7^ Very quickly, a reasonable “guesstimate” can be made for what initially seems like an impossible question.

In the paragraphs that follow, we will attempt our own Fermi Problem. Based on available data, the overarching question is “*How much Medicare reimbursement is lost annually to undercoding of outpatient visits in Florida and how might this impact clinical pharmacists*?” It is hoped that our approximation will bring greater attention to billing issues, which should be of great concern for clinical pharmacists striving to build sustainable service lines and potentially offset their salary via incident-to billing.

### 1.2 Prevalence of coding issues

Undercoding, overcoding, and miscoding is far more rampant than most healthcare professionals, including pharmacists, may realize.^8^ Undercoding or “downcoding” occurs when a billing provider intentionally codes a visit as less serious or extensive than it actually was. Conversely, overcoding or “upcoding” involves submitting codes for services that were never received or reporting a visit as more intensive than it actually was.^9^ Both practices are considered, by the letter of the law, to be fraud.^9^

The prevalence of such issues has received little attention outside the esoteric world of billing and coding, a subject where most pharmacists have little formal training. Several sources hint at how widespread they might be. First, according to a United States Department of Health and Human Services 2015 report, established office visits had a 35.5% rate of improper documentation and a 59.7% rate of incorrect coding.^10^ More specifically, internal medicine visits demonstrated a 14.8% improper payment rate and a 43.9% rate of incorrect coding, while 12.3% of family practice visits were improperly paid and 33.9% were incorrectly coded.^10^ Second, a study by Holt and colleagues (2010) found that one-third of reviewed family medicine visits were undercoded.^11^ Third, according to a 2005 survey by the American Academy of Professional Coders (AAPC), within any sample of 200 claims, 41% are overcoded and 45% are undercoded.^12^

While any sort of coding issue is problematic, perhaps the most pernicious is undercoding. In our experience, physicians and health system coders undercode to avoid insurance rejections or audits or even to make visits more affordable for patients.^8^^,^^9^^,^^12^ Though studies have found overcoding to be as commonplace as undercoding, we will focus on undercoding in the predictive model that follows since it correlates most directly with sacrificed revenue that could be used to support clinical pharmacy services.^8^

### 1.3 What is incident-to billing?

Medicare defines “incident-to” activities as “services furnished incident to physician professional services in the physician's office.”13., 14., 15 Within an “incident-to” practice model, outpatient visits are billed under Medicare Part B and paid according to the Centers for Medicare and Medicaid Services (CMS) Physician Fee Schedule. Though clinical pharmacists can perform any number of delegated tasks—customarily dictated in a collaborative practice agreement—it is the physician who ultimately bills Medicare for such services, not the pharmacist. To remain compliant, the physician must still perform an initial assessment, establish a diagnosis (e.g., atrial fibrillation), and develop a treatment plan (e.g., start warfarin 5 mg daily) before pharmacists may furnish clinical services.^15^ Pharmacists are eligible to receive partial or full financial credit when reimbursement is obtained, the terms of which may be outlined in a Memorandum of Understanding between pharmacists and physicians.^15^

Evaluation and management (E/M) services are customarily billed to Medicare using Current Procedural Terminology (CPT) codes. In brief, Level 1 services use code 99211, while 99,213 denotes Level 3 services and Level 4 services utilize 99,214. Level 3 and Level 4 services are generally reserved for more complicated patients, that is, those with multiple chronic conditions (i.e. ≥ 2 disease states).^16^ Medicare assigns each CPT code an average reimbursement value each year, which are provided in Table 1.^17^ To align more closely with state population estimates from 2019, 2018 reimbursement figures will serve as the foundation for our predictive model.

**Table 1:** Number of visits required for pharmacist to break even.^a^

Visit Frequency	Level 1	Level 3	Level 4
Average Medicare reimbursement (2018)	$21.96	$74.16	$109.44
Visits per year^b^	6374.00	1997.00	1355.00
Visits per week	122.60	38.40	26.10
Visits per day	24.50	7.70	5.22

Source: Medicare Physician Fee Schedule Look-Up Tool (https://www.cms.gov/medicare/physician-fee-schedule/search/overview).

a Assumes $0 overhead costs and a salary plus fringe of $125,000.

b Assumes 52 weeks per year.

As of January 2021, CMS has ruled that physicians cannot use CPT codes other than 99,211 when pharmacists provide incident-to care.^18^^,^^19^ By essentially capping the revenue potential of clinical pharmacists, this ruling jeopardizes their ability to create self-sustaining service lines and threatens the quality and continuity of care they provide. The number of outpatient visits needed for a clinical pharmacist to “break-even” has been published already.^15^ It has been reintroduced to illustrate the marked differences in reimbursement between each level of service (Table 1). It simply isn't feasible—from a clinical and financial standpoint—for most pharmacists to fully support themselves and provide high-quality care with Level 1 revenue alone.^15^ Since pharmacists are now capped at Level 1 reimbursement, it is more vital than ever that recognized billing providers code visits correctly. The additional revenue to be gained from proper coding could help to cover the costs of clinical pharmacists given their inability to generate significant revenue on their own. Ultimately, having clinical pharmacists involved in patient care improves quality, safety, and overall healthcare costs.

## 2 Methods

Several data sources were utilized in the creation of our revenue model. First, we drew upon the 2018 National Ambulatory Medical Care Survey (NAMCS), which provides nationally representative data for primary care or ambulatory care visits to physician offices within the United States.^20^ It is important to note that these data pertain strictly to non-federal office-based physician visits, not pharmacist-related visits. Instead, we used these office-based physician data as proxies to estimate (a) how many primary care or ambulatory care-related visits likely occur in Florida each year, (b) how many Medicare visits occur annually in Florida, (c) how many Medicare visits with established patients occur each year in the state, and (d) how many Medicare patients with two or more chronic conditions were eligible to be seen. It is these types of ambulatory encounters—involving patients with multiple chronic conditions and ultimately billed to Medicare—that were most eligible for incident-to pharmacist billing prior to 2021.

Based on data from the 2018 NAMCS, our model assumes the following:

(a) 136.6 primary care visits per 100 persons per year^⁎^.
(b) 29.1% Medicare or Medicare plus Medicaid visits^⁎⁎^.
(c) 91.5% of Medicare visits involve established patients^⁎⁎⁎^.
(d) 38.3% of patients have two or more chronic conditions^⁎⁎⁎⁎^.

*National Ambulatory Medical Care Survey - 2018 National Summary Tables. Table 1: Physician office visits, by selected physician characteristics: United States, 2018. Specialty type: primary care, Number of visits per 100 persons per year.

**National Ambulatory Medical Care Survey - 2018 National Summary Tables. Table 5: Expected sources of payment at office visits: United States, 2018. Medicare (27.7%), Medicare and Medicaid (1.4%).

***National Ambulatory Medical Care Survey - 2018 National Summary Tables. Table 8: Continuity-of-care office visit characteristics, by specialty type: United States, 2018. Established patients, Specialty type: Primary care.

**** National Ambulatory Medical Care Survey - 2018 National Summary Tables. Table 17: Presence of selected chronic conditions at office visit, by patient age and sex: United States, 2018: One or more chronic conditions: two (16.1%), three or more (22.7%).

These assumptions regarding pharmacist-related services are appropriate for our model for the following reasons. First, Medicare patients or Medicare plus Medicaid visits would be billed under incident-to services. Second, per incident-to billing rules, physicians must assess patients and develop a treatment plan before pharmacists may assist in their care.^15^ Therefore, only established office visits are eligible for clinical pharmacists. Moreover, several studies suggest that undercoding is more prevalent in visits for established patients.^8^ Third, patients with two or more chronic conditions and a greater level of medical complexity are more likely to be seen for Level 3 or Level 4 encounters.

Next, data pertaining to the ratio of Level 3 (99213) to Level 4 services (99214) have been established by CMS; according to their normalized ratio of 1.08 for Level 3-to-Level 4 services, one should expect the number of 99,213 to 99,214 visits in most practices to be relatively equal.^21^^,^^22^ Average Medicare reimbursement figures for 2018 were obtained from the Physician Fee Schedule Look-up Tool.^17^ Finally, the assumption that one-third of office visits are potentially undercoded is based on the work of Holt and colleagues (2010), but is supported by multiple studies.^8^^,^^11^ Due to the use of publicly available, de-identified primary care data, Institutional Review Board or Ethics Board approval was not required for this study.

## 3 Results

Using the aforementioned data points, we have generated the following predictive model outlining how much Medicare revenue is potentially lost each year by providers undercoding established outpatient visits (Table 2). It should be emphasized that these figures represent provider and non-physician provider visits, as opposed to strictly clinical pharmacist visits.

**Table 2:** Predictive Model for Medicare Billing Losses Due to Under-Coding.

PREDICTIVE MODEL FOR MEDICARE BILLING LOSSES		
Number of Florida residents (based on 2019 US census data)^23^	21,477,737	
Assumes 136.6 primary care visits per 100 persons per year^20^	29,338,589	Primary care visits per year in Florida
Assumes 29.1% Medicare or Medicare + Medicaid visits^20^	8,537,529	Medicare visits per year in Florida
Assumes 91.5% established patients^20^	7,811,839	Medicare visits involving established patients in Florida
Presence of One or More Chronic Conditions		
Patients with ≥2 chronic conditions (16.1%)^20^		
Patients with ≥3 chronic conditions (22.7%)^20^		
Total: 38.8% patients with two or more chronic conditions^20^	3,030,994	Potential Medicare patients with ≥2 chronic conditions
Medicare Incorrect Billing Costs		
Assumes 33.3% undercoding rate (established office visits)^11^	2,603,946	Under-coded Medicare office visits in Florida
Assumes roughly 50% split between Level 3 and Level 4 services^21^	1,301,973	Level 3 office visits undercoded
Assumes roughly 50% split between Level 3 and Level 4 services^21^	1,301,973	Level 4 office visits undercoded
Average Medicare Reimbursement by Visit Type (2018)^18^ • Level 1 = $21.96 • Level 3 = $74.16 • Level 4 = $109.44	*Billing for Level 3 instead of Level 4:* ($109.44 - $74.16) * 1,301,973 *Billing for Level 1 instead of Level 3:* ($74.16 - $21.96) * 1,301,973	
Estimated Losses Due to Provider Undercoding		
Billing for Level 3 instead of Level 4	$45,933,607	
Billing for Level 1 instead of Level 3	$67,962,990	
Projected annual losses due to provider undercoding:	$113,896,597	

This model suggests that of the 29.34 million projected primary care visits in the state of Florida each year, approximately 2.6 million (or 8.9%) are likely undercoded. This 8.9% undercoding rate is quite similar to the 8.49% improper payment rate for Part B providers mentioned by CMS' Comprehensive Error Rate Testing (CERT) program.^24^ Each year, this program reviews a statistically valid, stratified, randomized sample of Medicare claims to determine if they were paid properly under Medicare coverage, coding, and payment rules.^24^ If the approximately 1.3 million projected Level 3 visits were undercoded as Level 1 visits, it would lead to $67.96 million in sacrificed revenue. In addition, if the same number of Level 4 visits were billed as Level 3 instead, an estimated $45.93 million in revenue would be lost. In combination, this suggests that $113.9 million could be lost each year due to billing providers undercoding outpatient office visits within Florida.

## 4 Discussion

Though we have reason to believe—based on figures presented earlier—that coding issues are rather widespread, the answer to how much these practices truly cost healthcare practices, especially at the state level, remains murky.^8^^,^^25^ Ordinarily, it would be virtually impossible to answer since—to our knowledge—no single source collects, distills, and reports all of this information. Instead, one must stitch together data from several disparate sources. While our Fermi Problem merely serves as a rough estimate, it is rather alarming to see that nearly $114 million could be lost annually to undercoding in Florida alone. Many clinical pharmacist positions and many beneficial service lines could be underwritten with that lost revenue since, as of January 2021, pharmacists' work is strictly limited to Level 1 reimbursement. In principle, this lost revenue could support the work of hundreds of clinical pharmacists, leading to increased patient visits and improved clinical outcomes related to chronic disease state management.

Ultimately, what are the biggest dangers to habitual and widespread undercoding? First and perhaps most obvious of all, undercoding negatively impacts reimbursement for practices, providers, and team members like clinical pharmacists.26., 27., 28., 29., 30., 31. Simply put, coding drives revenue. Some sources assert that individual physician practices could sacrifice as much as $100,000 annually to undercoding.^13^ From a physician standpoint, coding could also drive personal compensation since it is often used as a proxy for their clinical productivity.^20^ From a clinical pharmacist standpoint, undercoding deprives them of additional revenue that could be used to cover the costs of their salaries, especially since CMS' ruling hinders their ability to generate meaningful revenue on their own. As was illustrated earlier, there is a substantial difference between the number of Level 1 and Level 4 visits required for clinical pharmacists to self-fund their clinical roles. Finally, patients are deprived of intensive, high-quality healthcare when clinical pharmacists are unable to financially support their service lines. Several studies illustrate that—on top of improvements in clinical outcomes—clinical pharmacists can save health plans hundreds—if not thousands—of dollars per patient per year when they are allowed to manage chronic disease states.^32^, ^33^, ^34^ Furthermore, it is estimated that for each dollar spent on clinical pharmacy services, more than $10 in healthcare spending is saved long-term.^35^

Despite the widespread adoption of electronic medical records (EMR), this technology has failed to mitigate coding and billing errors by physicians and practices. To date, there has been no visible improvement in E/M billing accuracy.^8^ Sources agree that most physicians are ill-prepared for billing and coding. Many residency programs fail to prepare graduates for the legal and financial responsibilities of this practice.^8^ Even highly experienced physicians are prone to poor documentation practices. Studies have found no relationship between a physician's years of coding and billing experience and their overall accuracy.^8^ Paradoxically, Zuber and colleagues (2000) found no statistical difference in accuracy between residents with 2.3 years of experience and their attending physicians with 23.3 years of experience.^36^ Moreover, Brennan and colleagues (2011) found that although physicians grasped the negative impact of billing and coding errors, billing systems and guidelines were so complex and cumbersome that little effort was made to improve accuracy.^37^ These issues are significant not only to physicians but clinical pharmacists as well since Medicare does not yet recognize pharmacists as billing providers.^38^^,^^39^ Until that occurs, pharmacists may have to rely on physician billing to bankroll their positions and, in the same token, must suffer when coding errors and reimbursement shortfalls occur.^15^ This provides an even greater incentive for pharmacists and professional organizations across the country to push for provider status and liberation from incident-to billing models.^38^^,^^39^

There are multiple limitations worth mentioning. First, this study was conducted using publicly available data for office-based physician visits, not pharmacist-related visits. To our knowledge, nationally representative office-based data for ambulatory pharmacist visits simply do not exist. Instead, we utilized what was known—in this case, primary care visits involving Medicare patients with multiple chronic conditions—to estimate what was unknown: the number of clinical encounters eligible for incident-to pharmacist billing in Florida. Second, since part of our revenue model (Table 2) was based on Florida's unique 2019 census data, projected annual losses due to provider undercoding in other states will almost certainly differ.

## 5 Conclusions

It is estimated that undercoding—or billing encounters for a lower level of service than may be justified—may occur in anywhere from 33 to 45% of outpatient visits. The prevalence and financial impact of this issue has received little attention outside the world of billing and coding. Practices and physicians engaging in undercoding sacrifice substantial reimbursement but, until now, the magnitude of what was lost at the state level remained unclear. Our model suggests that 2.6 million (or 8.9%) of the 29.34 million projected primary care visits in Florida are undercoded each year, which could lead to nearly $114 million in lost revenue. If our estimate is even reasonably accurate, the loss of such revenue undermines the long-term viability of service lines by clinical pharmacists since CMS has recently ruled that they cannot bill beyond Level 1 for incident-to services. The implications are profound: when pharmacists are financially excluded from the healthcare team, clinical outcomes and healthcare spending suffer enormously. Lost revenue could support the work of countless clinical pharmacists, leading to increased patient visits, improved clinical outcomes related to chronic diseases, and decreased healthcare spending.

## Statement of funding source

This research received no specific grant from any funding agency in the public, commercial, or not-for-profit sectors.

## Declaration of Competing Interest

Dietrich is a grant and honorarium recipient from Bristol Myers Squibb-Pfizer Alliance. Tenpas has no financial disclosures.
